# Feasibility and Safety of Sodium Glucose Cotransporter-2 Inhibitors in Adults with Heart Failure after the Fontan Procedure

**DOI:** 10.1155/2022/5243594

**Published:** 2022-12-09

**Authors:** Jun Muneuchi, Yuichiro Sugitani, Masaru Kobayashi, Hiroki Ezaki, Hiromu Yamada, Mamie Watanabe

**Affiliations:** Department of Pediatrics, Kyushu Hospital, Japan Community Healthcare Organization, Kitakyushu, Japan

## Abstract

Sodium glucose cotransporter-2 (SGLT-2) inhibitors have been widespread in patients with heart failure; however, there is little information regarding its feasibility and safety among patients after the Fontan procedure. We presented five adults after the Fontan procedure who were treated with SGLT-2 inhibitors. All patients reduced oedema and/or pleural effusion despite other conjunct medications were ineffective. Although we did not measure the urine volume in all patients, all patients themselves reported an increase in urinary output after the administration of a SGLT-2 inhibitor. In addition, administration of a SGLT-2 inhibitor resulted in weight loss (4/5), an increase in systemic oxygen saturation (4/5), an increase in serum albumin level (4/5), an increase in estimated glomerular filtration ratio (4/5), and a decrease in plasma brain natriuretic peptide level (4/5). Our case series supported the feasibility and safety of SGLT-2 inhibitors in patients with Fontan circulatory failure, although the exact changes in urinary output were unknown in all patients. Further investigation will be required to explore a diuretic effect by SGLT-2 in patients after the Fontan procedure.

## 1. Introduction

Future developing comorbidities, including heart failure, arrhythmias, protein-losing enteropathy, and liver disease, have been a concern in patients following the Fontan procedure. The Fontan circulation is characterized, not only by ventricular dysfunction owing to a functionally single ventricle but also by an increase in circulatory impedance and a decrease in circulatory capacitance owing to a lack of a subpulmonary ventricle. Regardless, the key sign of the Fontan circulatory failure is systemic congestion. Recent studies have suggested that sodium glucose cotransporter-2 (SGLT-2) inhibitors reduced the risk of hospitalization in patients with chronic congestive heart failure, irrespective of the presence of diabetes mellitus [1–3]. Although the precise pharmacological effects on heart failure remain unclear, a SGLT-2 inhibitor plays a role in natriuretic effect [4–6]. Therefore, this beneficial effect is posited to reduce systemic congestion in patients after the Fontan procedure. However, there is little information available in the literature regarding its feasibility and safety. We present a case series of five adults with heart failure who were treated with these agents long after the Fontan procedure.

## 2. Case Reports

### 2.1. Case 1

A 45-year-old male who had undergone the Fontan procedure was admitted due to oedema and dyspnoea on exertion. The patient had received enalapril, digoxin, furosemide, aspirin, and warfarin. However, he experienced 19 unexpected hospitalizations due to atrial fibrillation, resulting in exacerbated heart failure. Chest radiography on admission showed a bilateral pleural effusion. These findings suggest failed Fontan circulation. He was classified as New York Heat Association (NYHA) functional class III. After the administration of dapagliflozin, the urinary output increased, symptoms promptly disappeared, and he was subsequently discharged. During the follow-up of 4 months, there was no rehospitalization despite the occurrence of paroxysmal atrial fibrillation. No adverse events occurred.

### 2.2. Case 2

A 36-year-old male who had undergone the Fontan procedure was admitted due to oedema and dyspnoea on exertion. Due to the patient's existing comorbidities including hypertension, obesity, type 2 diabetes mellitus, cerebrovascular disease, and chronic kidney disease, there were 10 unexpected hospitalizations to date. He was classified as NYHA class III, receiving enalapril, digoxin, furosemide, spironolactone, aspirin, and apixaban. Urinalysis showed proteinuria and haematuria, while a renal biopsy revealed focal segmental glomerulosclerosis. Oral administration of empagliflozin was started for a sustained elevation of haemoglobin A1C level and subsequently caused increased urinary output and disappearance of oedema. Further urinalysis revealed that the proteinuria and haematuria disappeared. During the follow-up period of 24 months, he remained stable without unexpected rehospitalization. No adverse events occurred.

### 2.3. Case 3

A 35-year-old male who had undergone the Fontan procedure was admitted due to oedema of the lower extremities after viral infection. The patient was classified as NYHA class II with comorbidities including obesity, chronic kidney disease, type 2 diabetes mellitus, sleep apnoea syndrome, and Fontan-associated liver disease. To date, there have been two other unexpected hospitalizations. He had received carvedilol, enalapril, digoxin, furosemide, spironolactone, aspirin, and warfarin. Administration of dapagliflozin was started, and oedema in the lower extremities subsided following an increase in urinary output. During the follow-up of 16 months, he was stable in the outpatient clinic without rehospitalization. No adverse events occurred.

### 2.4. Case 4

A 24-year-old male after the Fontan procedure visited the outpatient clinic because of systemic oedema and headache. The patient had protein-losing enteropathy and plastic bronchitis due to Fontan circulatory failure and classified as NYHA class III. He had received carvedilol, digoxin, furosemide, spironolactone, tadalafil, aspirin, and warfarin. However, there were 29 unexpected hospitalizations due to these comorbidities. He had no history of hypertension, obesity, or diabetes. Urinary output increased with the commencement of dapagliflozin treatment, leading to improved clinical manifestations. During the follow-up of 3 months, there was no unexpected hospitalization except for hospitalization for a scheduled lower intestinal endoscopy. No adverse event occurred. Protein-losing enteropathy and plastic bronchitis were not improved.

### 2.5. Case 5

A 21-year-old male presented the deterioration of pleural effusion and oedema in the face and lower extremities after the Fontan procedure. The patient was classified as NYHA class III with comorbidities including chronic kidney disease, type 2 diabetes mellitus, and Fontan-associated liver disease. He had received *β*-blocker, enalapril, digoxin, furosemide, and warfarin. Urinalysis revealed neither proteinuria nor haematuria. The administration of dapagliflozin was initiated due to a sustained elevation of haemoglobin A1C levels. The increased urinary output caused by dapagliflozin reduced oedema. During the follow-up of 18 months, he was stable without rehospitalization and any adverse effects.

## 3. Discussion

In our case series (Table 1), three out of five patients initially received SGLT-2 inhibitors for glucose intolerance before the evidence of clinical trials were published. However, the agents improved cardiac manifestations as well as proteinuria and haematuria. Therefore, we prescribed a SGLT-2 inhibitor due to the development of heart failure despite conventional medical therapy in the other two patients as well. Although the usual dose of dapagliflozin was 10 mg, we cautiously prescribed an initial dose of 5 mg in 4 patients since clinical effects on patients with the Fontan circulation were still unclear. All patients had increased urinary output and reduced oedema and/or pleural effusion despite other conjunct medications were ineffective. Although we did not measure the urine volume in all patients, all patients themselves reported an increase in urinary output after the administration of a SGLT-2 inhibitor. In addition, administration of a SGLT-2 inhibitor resulted in weight loss (4/5), an increase in systemic oxygen saturation (4/5), an increase in serum albumin level (4/5), an increase in estimated glomerular filtration ratio (4/5), and a decrease in plasma brain natriuretic peptide level (4/5), which did not reach statistical significance due to the limited number of cases (Table 2). Furthermore, there was no unexpected rehospitalization due to the deterioration of heart failure and no adverse events, such as urinary tract infection, during the mean follow-up period of 12 months (ranging from 2 to 20 months) after the initiation of a SGLT-2 inhibitor. Unfortunately, we did not assess hemodynamic status including pulmonary arterial pressure and resistance by invasive methods, such as cardiac catheterization or indwelling central venous pressure monitoring.

The phenotype of Fontan circulatory failure is not well understood. Actually, in our case series, 3 patients had tolerable central venous pressure, and 3 patients had high cardiac index. Therefore, pathophysiologic interactions between the cardiovascular system and advanced noncardiovascular end-organ dysfunction are supposed to play major roles in Fontan circulatory failure [7, 8]. Fontan circulatory failure cannot be explained by the contemporary concept of heart failure according to ventricular ejection fraction, that is, heart failure as a result of a reduced or preserved ejection fraction [9]. The application of conventional guidelines for chronic heart failure should be carefully considered in patients with the Fontan circulatory failure. Some drugs that may be beneficial for some aspects of the Fontan circulation may be difficult to use because of adverse effects for another aspect of the Fontan circulation. In addition, targets for drug treatment in patients after the Fontan procedure may vary over time and between individual patients [9]. The benefits of SGLT-2 inhibitors include natriuresis and glucose-induced osmotic diuretics, which leads to a reduction in intraglomerular pressure and an increase in glomerular filtration ratio [6]. These beneficial effects on the kidney result in improvements in ventricular filling, ventricular wall stress, and myocardial energetics [10]. From this perspective, we believe that a SGLT-2 inhibitor has the potential to elicit maximum diuretic function in the Fontan circulation. Our case series supports the postulation that SGLT-2 inhibitors can reduce rehospitalization rate of patients, although the pharmacological impacts of these drugs on heart failure are still in the black box.

In conclusions, SGLT-2 inhibitors were tolerable without acute adverse effects in adults who undergone the Fontan procedure, although the exact changes in urinary output were unknown in all patients. Large prospective studies are needed to assess the efficacy and safety of SGLT-2 inhibitors in this population.

## Figures and Tables

**Table 1:** Summary of baseline characteristics in five patients after Fontan procedure who were treated with a SGLT-2 inhibitor.

Case	Sex	Age	Primary diagnosis	Prior procedures	Age at Fontan (years) Type of Fontan	Comorbidities	BMI (kg/m^2^)	CI (L.min^−1^.m^−2^)	CVP (mmHg)	EF (%)	Valve regurg.	Peak VO_2_ (%)	Times of unexpected hospitalization and reasons
1	Male	45	AVSD, MGA, PA, IVCD, left isomerism	AP shunt, TCPS, PMI	22 ECC	Heart failure PAf Chylothorax/ascites FALD	21.0	4.87 High CI	20	53	Mod AR Mod AVVR	—	19 (0.82/year) Heart failure PAf
2	Male	36	AVSD, MGA, PS, right isomerism	AP shunt	13 ECC	Heart failure Hypertension Obesity Diabetes CKD CNS hemorrhage	31.3	2.55 Low CI	11	53	Mild AVVR	58	10 (0.45/year) Heart failure Thrombosis Infection
3	Male	35	DORV, hypoplastic LV	PAB, BCPS	11 ECC	Heart failure Obesity DM CKD FALD	37.9	5.02 High CI	18	54	Mild AVVR	52	2 (0.08/year) Heart failure
4	Male	21	HLHS	Norwood, BCPS	1 ECC	Heart failure PLE PB	16.5	5.09 High CI	11	53	Mod AVVR	—	29 (1.52/year) Heart failure PLE/PB
5	Male	24	AVSD, MGA, PA, right isomerism	AP shunt, BCPS	2 ECC	DM CKD	24.0	2.32 Low CI	12	41	Mod AVVR	71	1 (0.04/year) Heart failure

Times of admission means unexpected admission due to not scheduled examination or treatment but cardiovascular symptoms. AP shunt: aortopulmonary shunt; AVSD: atrioventricular septal defect; BMI: body mass index; CI: cardiac index; CKD: chronic kidney disease; CNS: central nervous system; CVP: central venous pressure; DM: diabetes mellitus; DORV: double outlet right ventricle; ECC: extracardiac conduit; EF: ejection fraction; FALD: Fontan-associated liver disease; HLHS: hypoplastic left ventricle; IVCD: inferior vena cava defect; LV: left ventricle; MGA: malposition of the great arteries; PA: pulmonary atresia; PAB: pulmonary arterial banding; PAf: paroxysmal atrial fibrillation; PB: plastic bronchitis; PLE: protein-losing enteropathy; PMI: pacemaker implantation; PS: pulmonary stenosis; TCPS: total cavopulmonary shunt; Valve regurg.: valve regurgitation.

**Table 2:** Changes in clinical data before and after administration of a SGLT-2 inhibitor.

Case	SGLT-2 inhibitor	Duration of SGLT-2inhibitor administration (months)	Data acquisition timing	Weight (kg)	HR (min^−1^)	SO_2_ (%)	Mean BP (mmHg)	HbA1C (%)	Ht (%)	Platelet (×10^9^/L)	Albumin (g/dL)	Total bilirubin (mg/dL)	AST (IU/dL)	*γ*GTP (IU/dL)	Na (mmol/L)	Creatinine (mg/dL)	eGFR (mL/min/1.73m^2^)	BNP (pg/mL)
1	Dapagliflozin 5 mg	3	Before	55	85	83	86	5.6	43	0.93	3.3	4.6	50	155	140	1.02	64	193
			After	53	87	91	87	—	44	0.78	3.1	3.9	45	139	141	1.28	50	277
2	Empagliflozin 10 mg	20	Before	80	114	91	93	7.3	52	3.15	2.9	1.5	26	32	138	1.21	56	231
			After	85	83	92	90	7.7	56	3.30	3.4	0.5	48	51	139	1.12	60	7
3	Dapagliflozin 5 mg	12	Before	116	91	85	86	6.5	41	1.57	2.8	1.0	41	78	140	0.84	72	52
			After	106	83	89	85	6.3	53	1.18	3.8	2.7	45	138	140	0.97	140	57
4	Dapagliflozin 5 mg	2	Before	34	92	81	93	3.2	35	2.35	1.8	0.1	16	17	137	0.60	113	43
			After	33	94	79	94	—	32	2.97	2.0	0.1	17	17	132	0.74	137	38
5	Dapagliflozin 5 mg	14	Before	69	103	88	84	7.8	60	1.64	2.9	1.7	55	108	125	1.52	72	101
			After	66	75	92	81	6.6	62	1.57	4.4	2.6	39	62	141	1.07	125	12
*P* value				0.345	0.224	0.138	0.224	—	0.224	0.892	0.079	0.715	0.892	0.224	0.465	0.685	0.500	0.345

*P* value < 0.05 means statistical significance when clinical data were compared before and after administration of a sodium glucose cotransporter-2 inhibitor using the Wilcoxon signed rank test. AST: aspartate aminotransferase; BNP: brain natriuretic peptide; BP: blood pressure; *γ*GTP: gamma-glutamyl transpeptidase; HbA1C: haemoglobin A1C; HR: heart rate; Ht: haematocrit; eGFR: estimated glomerular filtration ratio; SGLT-2 inhibitor: sodium glucose cotransporter-2 inhibitor; SO_2_: systemic oxygen saturation.

## Data Availability

The clinical data used to support the findings of this study are included within the article.
